# Hypertension secondary to nitric oxide depletion produces oxidative imbalance and inflammatory/fibrotic outcomes in the cornea of C57BL/6 mice

**DOI:** 10.1007/s13105-022-00916-2

**Published:** 2022-08-09

**Authors:** Álvaro Santana-Garrido, Claudia Reyes-Goya, Ana Arroyo-Barrios, Helder André, Carmen M. Vázquez, Alfonso Mate

**Affiliations:** 1 Departamento de Fisiología, Facultad de Farmacia, Universidad de Sevilla, 41012 Seville, Spain; 2 Epidemiología Clínica y Riesgo Cardiovascular, Instituto de Biomedicina de Sevilla (IBIS), Hospital Universitario Virgen del Rocío/Consejo Superior de Investigaciones Científicas/Universidad de Sevilla, 41013 Seville, Spain; 3 Department of Clinical Neuroscience, St. Erik Eye Hospital, Karolinska Institutet, 11282 Stockholm, Sweden

**Keywords:** L-NAME hypertension, Intraocular pressure, Cornea, Fibrosis, Inflammation, Oxidative stress

## Abstract

Arterial hypertension (AH) leads to oxidative and inflammatory imbalance that contribute to fibrosis development in many target organs. Here, we aimed to highlight the harmful effects of severe AH in the cornea. Our experimental model was established by administration of NG-nitro-L-arginine-methyl-ester (L-NAME) to C57BL/6 mice, which were monitored weekly for arterial blood pressure and intraocular pressure (IOP). Morphological studies of ocular tissues were accompanied by analyses of reactive oxygen species generation, and localization/expression of NAPDH oxidase isoforms (NOX1, NOX2, NOX4) and inflammatory biomarkers (PPARα, PPARγ, IL-1β, IL-6, IL-10, TNF-α, and COX-2). Masson’s trichrome and Sirius Red staining were used to explore the fibrotic status of the cornea. The expression of collagen isoforms (COL1α1, COL1α2, COL3α1, COL4α1, COL4α2) and relevant metalloproteinases (MMPs) and their tissue inhibitors (TIMPs) were also quantified to evaluate the participation of collagen metabolism in AH-related corneal damage. Hypertensive animals showed an increase in IOP values, and a thinner cornea compared with normotensive controls. Moreover, AH increased NADPH oxidase activity and reactive oxygen species generation in the cornea, which was accompanied by transcriptional upregulation of NOX isoforms and inflammatory biomarkers, while reducing PPAR expression. L-NAME-treated animals also developed corneal fibrosis with overexpression of collagen isoforms and reduction of factors responsible for collagen degradation. This is the first study reporting structural changes in the cornea and elevated IOP in L-NAME-treated mice. Overexpression of the NADPH oxidase system and collagen deposition might play a substantial role in the pathogenic mechanisms contributing to ocular disturbances in a context of severe hypertension.

## Introduction

Arterial hypertension (AH) is a systemic disease considered as one of the major risk factors for the development and progression of cardiovascular, renal, and neurological diseases, among others [61]. With a global prevalence of around 30–45%, AH is defined as a sustained rise of systolic blood pressure (SBP) and/or diastolic blood pressure (DBP) values over 140/90 mmHg, respectively, with different hypertension grades depending on the precise values [59]. Despite the potential and well-documented harmful effects of hypertension on the eye, specifically in the retina and choroid [16], additional outcomes in the cornea in this context remain unexplored.

Oxidative stress, defined as an excessive production of reactive oxygen species (ROS), is known to produce tissue damage, cell death, and acceleration of the aging process. In turn, inflammation is a complex process aiming to protect the tissues from damage and infectious agents [52]. Both processes contribute to the pathogenesis of various ocular diseases, including not only the development of retinopathies but also corneal and conjunctival disorders, such as dry eye syndrome, pterygium, or keratoconus [40].

Regarding oxidative stress, many reports support that, in a context of AH, the enzyme nicotinamide adenine dinucleotide phosphate (NADPH) oxidase (NOX) is a major enzymatic source for ROS production (mainly superoxide anion (O_2_^.−^) and/or peroxide hydrogen (H_2_O_2_)), with subsequent organ damage [31]. The expression of different NOX isoforms (NOX1-5 and Duox1-2) has been found in various tissues with differential functions [70]. Although NAPDH oxidase seems to be highly implicated in the oxidative imbalance induced by AH in the retina [48, 49], no link between AH and oxidative stress has been established so far in the cornea. Along with the rest of the ocular surface, and due to continuous exposure to environmental oxidizing agents, the cornea has an effective antioxidant defense mechanism. However, in some circumstances, an oxidative imbalance develops that favors the pathogenesis of corneal diseases and can even lead to blindness [38]. Therefore, it is important to investigate potential mechanisms capable of altering the oxidative status of the cornea.

The oxidative imbalance generated by overproduction of ROS can lead to inflammatory processes, thus playing a key function in the pathophysiology of many diseases. The low-grade inflammation involved in AH modulates the expression of the main inflammatory biomarkers, including tumor necrosis factor alpha (TNF-α), cyclooxygenase 2 (COX-2), and some interleukin isoforms [57]. In relation to eye diseases, corneal inflammation has been well associated with the development and progression of dry eye disease and keratoconus [34, 64]. Along with fibrosis, the importance of inflammation has been studied in corneal pathological events, such as corneal wounds [66]. However, in spite of the importance of AH in inflammatory and fibrotic ocular events, e.g., in retinopathies, its possible pathological role in the cornea is still unknown.

Fibrogenic processes may be aggravated by inflammatory and oxidative events in AH. Arterial fibrosis and extracellular matrix (ECM) deposition related to AH contribute to hypertensive target organ damage, possibly because AH leads to a modification of the turnover of ECM components [56]. ECM components, among which collagen is especially relevant, provide structural integrity to the cornea for proper functionality. A balanced composition of collagen fibers at the corneal stroma (which constitutes nearly 90% of the corneal thickness) is very important to maintain its transparency [35]. Type I collagen is the main collagen isoform in the cornea, accompanied by type III and type IV collagen fibers in smaller amounts. Fibrotic processes in the cornea, including wound healing and inflammation, have been associated with an upregulation of collagens that can alter the transparency of cornea and produce visual loss [33]. Maintenance and repair of the corneal ECM are coordinated by a brittle balance between matrix metalloproteinases (MMPs) and its tissue inhibitors (TIMPs), which are altered in some corneal disorders [46]. Maintaining this balance is so important that therapies based on corneal collagen administration have been approved for treatment of corneal ecstatic disorders [14]. Surprisingly, despite the importance of collagen metabolism for an adequate corneal function, the implication of AH at this level has not been studied yet.

To investigate the possible role of AH in the development and progression of corneal pathologies, here, we measured different biomarkers of oxidative stress, inflammation, and fibrosis in a mice model of AH induced by administration of NG-nitro-L-arginine-methyl-ester (L-NAME, an inhibitor of nitric oxide (NO) synthesis). Blood pressure and intraocular pressure (IOP) monitoring, morphometric analyses of the cornea, measurement of ROS and NO levels, and the activity and location/expression of NOX components, inflammatory biomarkers (PPARα, PPARγ, TNF-α, COX-2, IL-1β, IL-6, and IL-10), and fibrosis-related parameters (including collagen profile and MMP/TIMP enzymes) were assayed in the cornea of L-NAME-treated mice and compared with a parallel normotensive control group.

## Materials and methods

### Animal characteristics and blood pressure

The experimental design was conducted in accordance with the European Union (EU) Directive 2010/63/EU and the National (RD 53/2013) guidelines for the care and use of Laboratory animals and was approved by the competent Institutional Animal Care and Use Committee (approval reference #13/03/2019/031, issued by Junta de Andalucía, Dirección General de la Producción Agrícola y Ganadera). Male C57BL/6 mice aged 10–12 weeks were obtained from the Center for Animal Production and Experimentation at the University of Seville (Spain). Mice were housed with free access to food and drink and exposed to 12:12-h light/dark, and randomly assigned into two groups: (1) normotensive mice fed a standard pellet diet (control group), and (2) hypertensive mice (via administration of 45 mg L-NAME/kg body weight/day in the drinking water) fed the same commercial diet (L-NAME group). The concentration of L-NAME in the water bottles was calculated weekly considering the evolution of body weight and water intake, and treatment was maintained for 6 weeks.

SBP and DBP were recorded weekly by the noninvasive tail-cuff technique with a pressure recorder (NIPREM 645, Cibertec, Spain). Values were estimated as the average of 3–4 consecutive records.

### Intraocular pressure measurements

Intraocular pressure (IOP) was measured using a rebound tonometer (Tonolab, Icare), following protocols described previously [58]. Briefly, tonometry was performed in awaked and unrestrained mice between 8:00 and 11:00 a.m. to minimize the effects of the circadian rhythm on IOP. IOP readings were obtained at the beginning (week 1), middle (week 3), and at the end (week 6) of the treatment with L-NAME. Mice were habituated to the tonometer from the week before the start of the measurements, and the readings were taken on the central cornea of both eyes.

### Cornea isolation and homogenization

Mice were euthanized by cervical dislocation. Corneas were rapidly dissected under a binocular stereoscopic microscope, snap-frozen in liquid nitrogen, and stored at − 80 °C until measurement of NAPDH oxidase activity. Fifty millimolar PBS (pH 7.4) containing protease inhibitors (Sigma Aldrich-Roche, Madrid, Spain) was used to obtain cornea homogenates (which included the two pooled corneas from each mouse) using a Potter–Elvehjem tissue grinder. Homogenates were centrifuged for 10 min at 10,000 × g and the supernatants were recovered to determine the protein concentration by the Bradford method.

### NADPH oxidase activity measurements

NADPH oxidase activity was measured in cornea homogenates by lucigenin-enhanced chemiluminescence, following routine protocols in our laboratory [48]. Potential sources of superoxide anion (O_2_^.−^) production in corneal samples were discriminated at 37 °C after a 5-min preincubation with 0.1 mmol/L DPI, oxypurinol, or rotenone (respective inhibitors of flavoproteins, xanthine oxidase, and mitochondrial electron transport chain; Sigma-Aldrich, Madrid, Spain). Similar protocols were followed when using an inhibitor of NOX1/4 (0.1 µmol/L GKT136901; Sigma-Aldrich, 492,000), specific NOX1 inhibitor (0.5 µmol/L ML171; Sigma-Aldrich, 175,226), and the pan-NADPH oxidase inhibitor (10 µmol/L VAS2870; Sigma-Aldrich, 5,340,320,001) to determine the relative contribution of each NOX isoform in total O_2_^.−^ production. All measurements were referred to the samples’ protein content, and results were always expressed as relative to those in the control group.

### Nitric oxide concentration

The concentration of NO in corneal homogenates was estimated following the Griess method [32]. The readings were normalized to the corresponding protein content of each sample and the results were plotted relative to the control group.

### Real-time PCR

RNA isolation from corneal tissue was performed using TRIzol® RNA isolation method (Thermo Fisher Scientific, Madrid, Spain) with the two pooled corneas from each animal. Reverse transcription and polymerase chain reactions were run as previously described [50] and using specific primers listed in Table 1, which were designed using the application PerlPrimer v. 1.1.20 (Marshall OJ). The integrity of isolated RNA and PCR products was confirmed by observing the appearance and quality of the relevant bands on agarose gels and determining A260/280 and A260/230 ratios in a NanoDrop (Thermo Fisher Scientific).

**Table 1:** Primers used for real-time PCR

Gene	Forward primer (5′ → 3′)	Reverse primer (5′ → 3′)	Accession number
Nox1	GGTTGGGGCTGAACATTTTTC	TCGACACACAGGAATCAGGAT	NM_172203.2
Nox2	CCCTTTGGTACAGCCAGTGAAGAT	CAATCCCACGTCCCACTAACATCA	FJ168469.1
Nox4	ATCACAGAAGGTCCCTAGCA	TAACCATGAGGAACAATACCAC	AF276957.1
TNF-α	CCACGCTCTTCTGTCTACTG	ACTTGGTGGTTTGCTACGAC	D84199.2
COX-2	ACCCCCTGCTGCCCGACACCT	CCAGCAACCCGGCCAGCAATC	NM_011198.4
IL1β	CCGTGGACCTTCCAGGATGA	GGGAAGGTCACACACCAGCA	M15131.1
IL6	CTCTGCAAGAGACTTCCATCC	TTCTGCAAGTGCATCATCGT	DQ788722.1
IL10	CTGGACAACATACTGCTAACCG	GGGCATCACTTCTACCAGGTAA	BC120612.1
COL1α1	GCTCCTCTTAGGGGCCACT	CCACGTCTCACCATTGGGG	NM_007742.4
COL1α2	GGTGAGCCTGGTCAAACGG	ACTGTGTCCTTTCACGCCTTT	NM_007743.3
COL3α1	CCTGGCTCAAATGGCTCAC	GACCTCGTGTTCCGGGTAT	NM_009930.2
COL4α1	CTAACGGTTGGTCCTCACTG	CGTGGGCTTCTTGAACATCTC	NM_009931.2
COL4α2	GGCTTCATCAAAGGAGTCAAGG	CCCAATGTCACCAAAGTCCC	NM_009932.4
MMP2	CAAGTTCCCCGGCGATGTC	TTCTGGTCAAGGTCACCTGTC	NM_008610.3
MMP3	GTCTTTGAAGCATTTGGGTTTCTC	GGTGTCATCCATAGCTCCTG	NM_010809.2
MMP9	CTGGACAGCCAGACACTAAAG	CTCGCGGCAAGTCTTCAGAG	NM_013599.5
TIMP1	CCAGAGCAGATACCATGATGG	CCACAGAGGCTTTCCATGAC	NM_001044384.1
TIMP2	GAATCCTCTTGATGGGGTTG	CGTTTTGCAATGCAGACGTA	NM_011594.3
TIMP3	TAGACCAGAGTGCCAAAGGG	CCAGGATGCCTTCTGCAAC	NM_011595.2
GAPDH	GCCAAAAGGGTCATCATCTCCGC	GGATGACCTTGCCCACAGCCTTG	XM_017321385.2

### Western blotting

Aliquots of corneal homogenates containing equal amounts of proteins (20 μg) were used to perform Western blot experiments following protocols previously described [48]. The primary antibodies used are listed in Table 2. Quantitative optical densitometry (Cytive Europe GmbH, Barcelona, Spain) was performed using β-actin as a loading control.

**Table 2:** Antibodies used for Western blotting analyses

1st antibody	Origin	Dilution	2nd antibody	Dilution	Reference
Anti-iNOS	Mouse monoclonal	1:1000	Goat Anti-Mouse	1:2000	sc-7271, Santa Cruz Biotechnology, Santa Cruz, CA
Anti-Nitrotyrosine	Mouse monoclonal	1:2000	Goat Anti-Mouse	1:3000	sc-32757, Santa Cruz Biotechnology,
Anti-NOX1	Mouse monoclonal	1:2000	Goat Anti-Mouse	1:3000	sc-518023, Santa Cruz Biotechnology,
Anti-NOX2	Rabbit monoclonal	1:7000	Goat Anti-Rabbit	1:8000	ab129068, Epitomics-Abcam, Burlingame, CA
Anti-NOX4	Rabbit monoclonal	1:7000	Goat Anti-Rabbit	1:8000	ab133303, Epitomics-Abcam
Anti-PPARα	Mouse monoclonal	1:1000	Goat Anti-Mouse	1:2000	sc-398394, Santa Cruz Biotechnology,
Anti-PPARγ	Mouse monoclonal	1:1000	Goat Anti-Mouse	1:2000	sc-271392, Santa Cruz Biotechnology,
Anti-β-actin	Mouse monoclonal	1:10,000	Goat Anti-Mouse	1:20,000	sc-47778, Santa Cruz Biotechnology,

### Histological sections and staining

Corneal tissue sections (5 μm) were obtained following a protocol previously reported by Santana-Garrido et al. [48]. Paraffin-embedded sections were used for morphometric analysis, Masson’s trichrome, Sirius Red staining, DHE staining, and immunofluorescence, as detailed below. Deparaffinized hematoxylin/eosin-stained sections (H/E) were photographed using an Olympus BX41 microscope coupled to an Olympus DP73 camera. The thickness of the corneal layers was always measured at the central height of the cornea using ImageJ-NIH freeware (v. 2.0.0). Furthermore, collagen fibers in the corneas were visualized using Masson’s trichrome (MTS; Merk, Germany, 1.00485) and picro Sirius Red (SRS; Sigma-Aldrich Inc, St Louis, USA) staining. MTS and SRS pictures were quantified using Image J-NIH freeware (v. 2.0.0) and following protocols detailed elsewhere [17].

### Corneal ROS measurements

Paraffin-embedded eye sections were used to estimate corneal ROS production in situ by dihydroethidium staining (DHE; MedChemExpress, Madrid, Spain, Cat. No. HY-D0079), following a protocol previously used in our laboratory [48]. The specificity of DHE staining in deparaffinized sections was evaluated by preincubation with 100 U/mL polyethylene glycol-conjugated superoxide dismutase (PEG-SOD; Sigma Aldrich, S9549) for 30 min at 37 °C. Parallel deparaffinized corneal sections were preincubated with specific NOX inhibitors VAS2870, GKT136901, and ML171 for 30 min at 37 °C. The slides were then mounted with DAPI Fluoromount-G® (SouternBiotech Associates, Inc, Birmingham, AL; Cat. No. 0100–20) after 20 min incubation with DHE at 37 °C. An Olympus DP73 fluorescence microscope (Tokyo, Japan) and Image J-NIH freeware (v. 2.0.0) were used to measure the intensities of the staining. Central corneal sections from normotensive and hypertensive animals were processed in parallel under similar conditions. Images were taken using the same exposure time and a × 10 objective. Results were expressed as relative to the control group.

### Immunohistofluorescence

The corneal expression of NOX and PPAR isoforms was evaluated by immunohistofluorescence in deparaffinized eye sections. Diva Decloaker (Biocare Medical, LLC, Pacheco, CA), an antigen retrieval reagent, and the following specific primary antibodies were used for immunostaining: mouse monoclonal anti-NOX1 (Santa Cruz Biotechnology, Santa Cruz, CA; Cat. No. sc-518023; 1:200 dilution); rabbit monoclonal anti-NOX2 (Epitomics-Abcam, Burlingame, CA; Cat. No. ab129068; 1:100 dilution); rabbit monoclonal anti-NOX4 (Epitomics-Abcam; Cat. No. ab133303; 1:500 dilution); mouse monoclonal anti- PPARα (Santa Cruz Biotechnology; Cat. No. sc-398394; 1:200 dilution); mouse monoclonal anti-PPARγ (Santa Cruz Biotechnology; Cat. No. sc-271392; 1:200 dilution). Goat anti-rabbit Alexa Fluor® 555 (Cohesion Biosciences Ltd., London, UK; Cat. No. CSA3411) and Goat anti-mouse Alexa Fluor® 647 (Cat. No. CSA3808) were used as fluorescent secondary antibodies, where appropriate, and sections were mounted with DAPI Fluoromount-G®. Image J-NIH freeware (v. 2.0.0) was used to measure the intensity of the staining on parallel images of both animal groups, which were acquired from central corneal sections and processed with the same settings. Results were expressed as relative to the control group.

### Statistical analyses

Data are presented as means ± SEM. Unless more than two groups were compared (where one-way ANOVA followed by Tukey multiple comparison test was performed), results were subjected to unpaired Student’s *t*-test using the GraphPad InStat software (San Diego, CA) and were considered significant at *p* < 0.05.

## Results

### Hypertensive general data and NO concentration

**Fig. 1:**
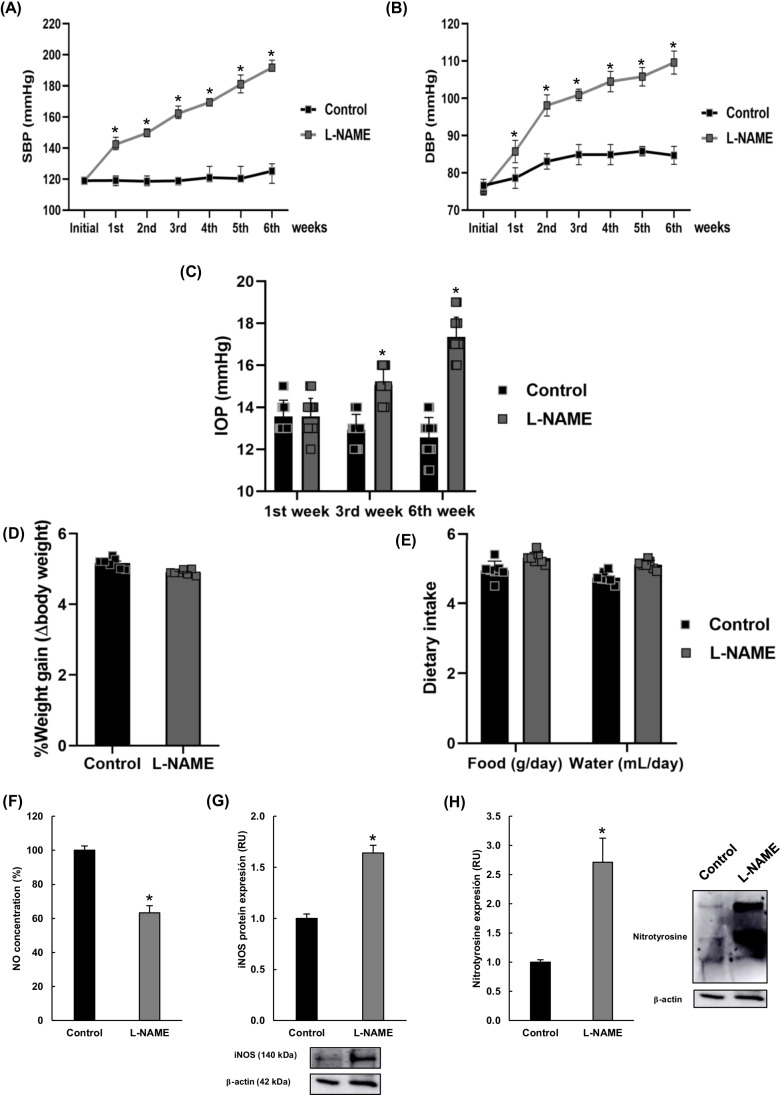
Animal general characteristics. Evolution of (**A**) systolic blood pressure (SBP), (**B**) diastolic blood pressure (DBP), and (**C**) intraocular pressure (IOP) values measured by rebound tonometry, in normotensive (control, black squares) and hypertensive (L-NAME, gray squares) animals throughout the 6-week experimental period. **D** Weight gain and **E** water/food intake at the end of the treatment. **F** Nitric oxide concentration, **G** iNOS protein expression, and **H** protein nitrosylation in corneal homogenates. Values in **F**–**H** are presented as relative to those of the control group, and all values are expressed as mean ± SEM of seven animals per group. *p < 0.05 vs control. RU relative units

As expected, elevated SBP and DBP values were found in the L-NAME group (196/104 mmHg, respectively) at the end of the 6-week treatment (Fig. 1A–B). Remarkable differences with the control group were found from the first week of L-NAME administration. As shown in Fig. 1C, IOP values (which showed no interocular variations) also increased during treatment with L-NAME; significant differences were observed from mid-treatment and became substantial at the end of the experimental period (13 vs 17 mmHg in control and L-NAME groups, respectively). On the other hand, the intake of L-NAME did not affect the animals’ weight gain or their drinking and feeding behaviors throughout the treatment (Fig. 1D–E).

The estimated concentration of NO in corneal homogenates was reduced by 40% in L-NAME-treated animals compared to the control group (Fig. 1F). This alteration was accompanied by an upregulation of the inducible isoform of nitric oxide synthase, iNOS (Fig. 1G). As an additional marker of oxidative stress, the degree of nitrosylated proteins was also quantified in corneal homogenates, showing a significant overexpression of 3-nitrotyrosine residues in NO-depleted mice (Fig. 1H).

### Arterial hypertension induces histomorphometric changes and ROS production in the cornea

**Fig. 2:**
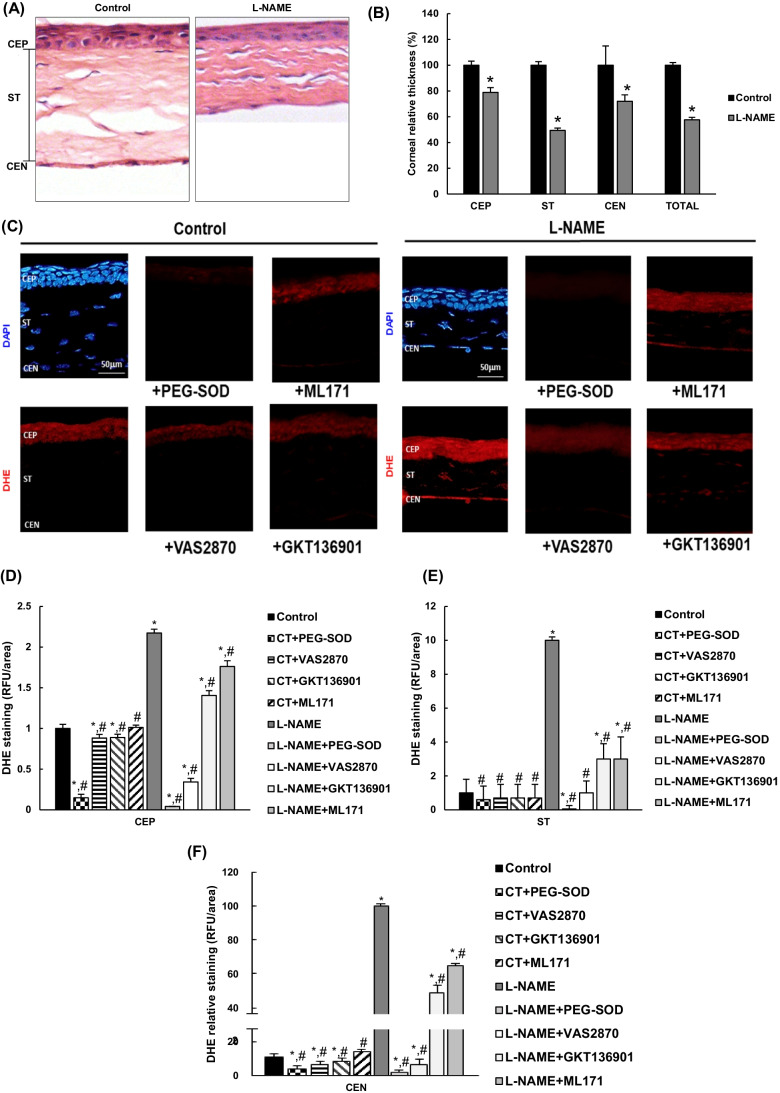
Histomorphometric analyses and ROS measurements in the cornea. **A** Representative images of hematoxylin/eosin staining and **B** corneal layer thickness in normotensive (control) and hypertensive (L-NAME) mice. **C** Reactive oxygen species (ROS) production in the cornea visualized by DHE labeling (red color) with or without preincubation with specific NOX inhibitors: NOX pan-inhibitor (VAS2870), dual NOX1/NOX4 inhibitor (GKT136901), and NOX1 inhibitor (ML171). The specificity of DHE staining was confirmed by preincubation with 100 U/mL polyethylene glycol-conjugated superoxide dismutase (PEG-SOD). Corneal layers can be distinguished with 4′,6-diamidino-2-phenylindole (DAPI, blue color) nuclei staining. **D**–**F** Relative fluorescence intensity in **C** relative to that of the control group and quantified using the Image J software. Magnification: × 10. ANOVA followed by Tukey multiple comparison test was performed to analyze the results, and values are expressed as mean ± SEM of seven animals per group. *p < 0.05 vs control; #*p* < 0.05 vs L-NAME. CEN, corneal endothelium; CEP, corneal epithelium; RFU, relative fluorescence units; ST, stroma

Figure 2A shows representative corneal images stained with H/E. Morphometric analysis revealed a noticeable reduction in the thickness of the corneal layers (about 42%) in L-NAME-treated animals (Fig. 2B). Specifically, this group presented significant reductions of 21%, 51%, and 28% in the corneal epithelium (CEP), stroma (ST), and corneal endothelium (CEN), respectively, compared with the equivalent layers of the control group.

Representative images of DHE-stained corneas are shown in Fig. 2C. DHE signal was intensified in L-NAME-treated mice in CEP (approximate twofold increase; Fig. 2D) and especially in ST and CEN layers (10- and 100-fold increases, respectively; Fig. 2E–F), because the staining was hardly visible in the latter in normotensive animals. To clarify the relative contribution of NOX isoforms to corneal ROS production, additional sections from control and L-NAME-administered mice were preincubated with different NOX inhibitors. The pan-inhibitor VAS2870 induced a major reduction of DHE-dependent O_2_^.−^ signal in the cornea of hypertensive mice (83%, 90%, and 99% in CEP, ST, and CEN, respectively, compared to the L-NAME group). Smaller reductions in DHE staining were observed in corneas of L-NAME-treated mice after incubation with the dual NOX1/NOX4 inhibitor, GKT136901 (36%, 70%, and 51% in CEP, ST, and CEN, respectively), or with the inhibitor of NOX1, ML171 (19%, 70%, and 35%, respectively). In turn, the inhibitory action on DHE-related baseline signals detected in normotensive mice was much lower. Taken together, these results suggest a higher release of ROS in corneas with a preferential role for NADPH oxidase isoforms NOX2 and NOX4, and also regional differences with the CEN being more affected than the CEP and the ST.

### NADPH activity and NOX isoforms are upregulated in hypertensive corneas

**Fig. 3:**
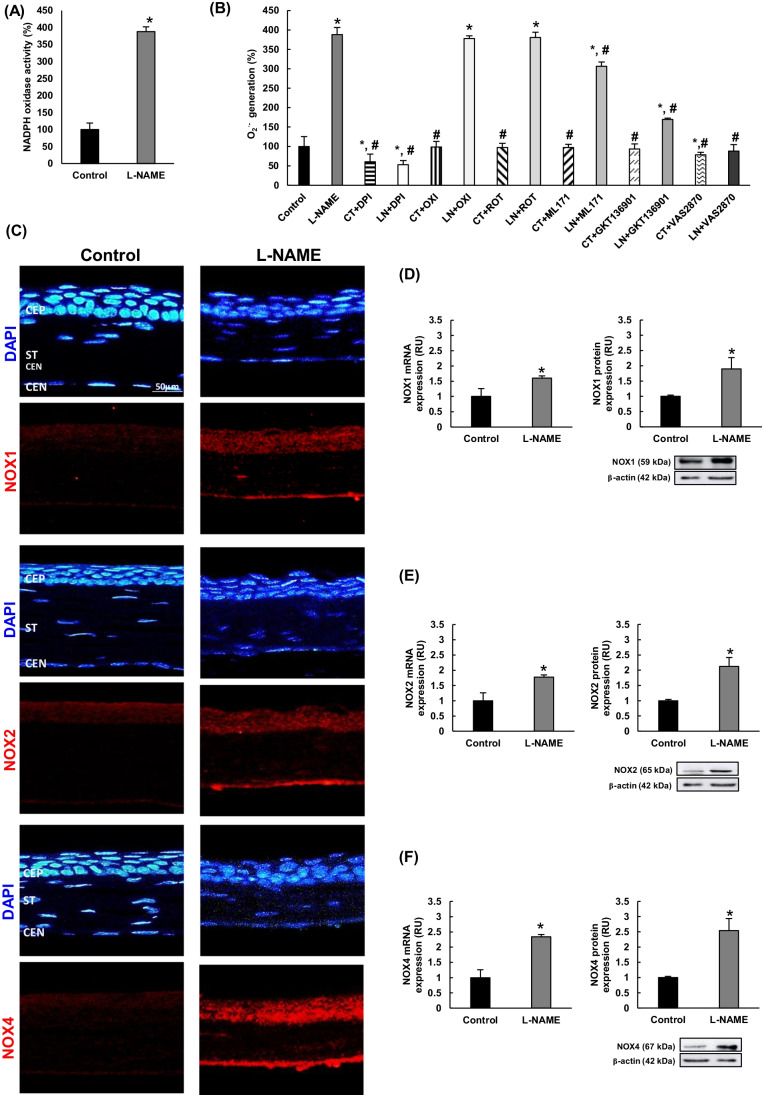
NADPH oxidase activity and NOX expression in the cornea. **A** Relative NADPH oxidase activity in cornea homogenates and **B** characterization of the primary source of superoxide anions after preincubation with different inhibitors: DPI (diphenyleneiodonium chloride, inhibitor of flavoproteins); OXI (oxypurinol, xanthine oxidase inhibitor); ROT (rotenone, inhibitor of mitochondrial electron transport chain); VAS2870 (NOX pan-inhibitor); GKT136901 (dual NOX1/NOX4 inhibitor); and ML171 (NOX1 inhibitor). **C** NOX expression (red color) and nuclei staining with DAPI (blue color) of NOX1 (top), NOX2 (middle), and NOX4 (bottom) in corneal layers from each experimental group. Magnification: × 10. mRNA and protein expression of (**D**) NOX1, (**E**) NOX2, and (**F**) NOX4 in cornea homogenates from normotensive (control) and hypertensive (L-NAME) mice. Quantitative fold changes in gene expression were determined relative to the corresponding value for the housekeeping gene GAPDH. ANOVA followed by Tukey multiple comparison test was performed to analyze the results depicted in **B**. Values are expressed as mean ± SEM of seven animals per group. *p < 0.05 vs control; ^#^*p* < 0.05 vs L-NAME. CEN, corneal endothelium; CEP, corneal epithelium; RU, relative units; ST, stroma

To confirm the pivotal role of the NADPH oxidase system in local O_2_^.−^ production, corneal homogenates were obtained and used to measure NADPH oxidase activity and to check the source(s) of O_2_^.**−**^ generation locally in this tissue. A significant (fourfold) increase in the activity of NADPH oxidase was found in the L-NAME group relative to the control group (Fig. 3A). Corneal O_2_^.−^ production in hypertensive mice remained unaltered when homogenates from these animals were preincubated with either oxypurinol or rotenone, while DPI abolished superoxide overproduction completely (Fig. 3B). Also shown in Fig. 3B, preincubation with NOX inhibitors reduced superoxide production in L-NAME animals by 21%, 56%, and 77% when ML171 (NOX1-inhibitor), GKT136901 (dual NOX1/NOX4 inhibitor), and VAS2870 (NADPH oxidase pan-inhibitor) were used, respectively. On the other hand, preincubation with these inhibitors had essentially no effect on corneal homogenates obtained from normotensive animals, except for DPI and VAS2870 (40% and 22% reductions, respectively).

Isoforms NOX1, NOX2, and NOX4 were detected in corneal sections with a higher immunofluorescence signal in CEP, ST, and CEN from L-NAME-administered animals (Fig. 3C). The most remarkable differences between normotensive and hypertensive animals were found at the CEN level (especially for NOX4 signal). At the ST, NOX-related signals were weak in L-NAME animals and absent in the control group. In addition, the results of gene and protein expression confirmed the upregulation of these three NOXes in the L-NAME group; thus, our experimental model of hypertension by nitric oxide deprivation presented with significant increases in the mRNA and protein expression of NOX1, NOX2, and NOX4 (~ 2–2.5-fold; Fig. 3D–F). Collectively, our results indicate that NOX4 might be the main isoform of NADPH oxidase involved in excess O_2_^.−^ release at the corneal level in hypertensive mice.

### Profile of inflammatory biomarkers in L-NAME-hypertensive corneas

**Fig. 4:**
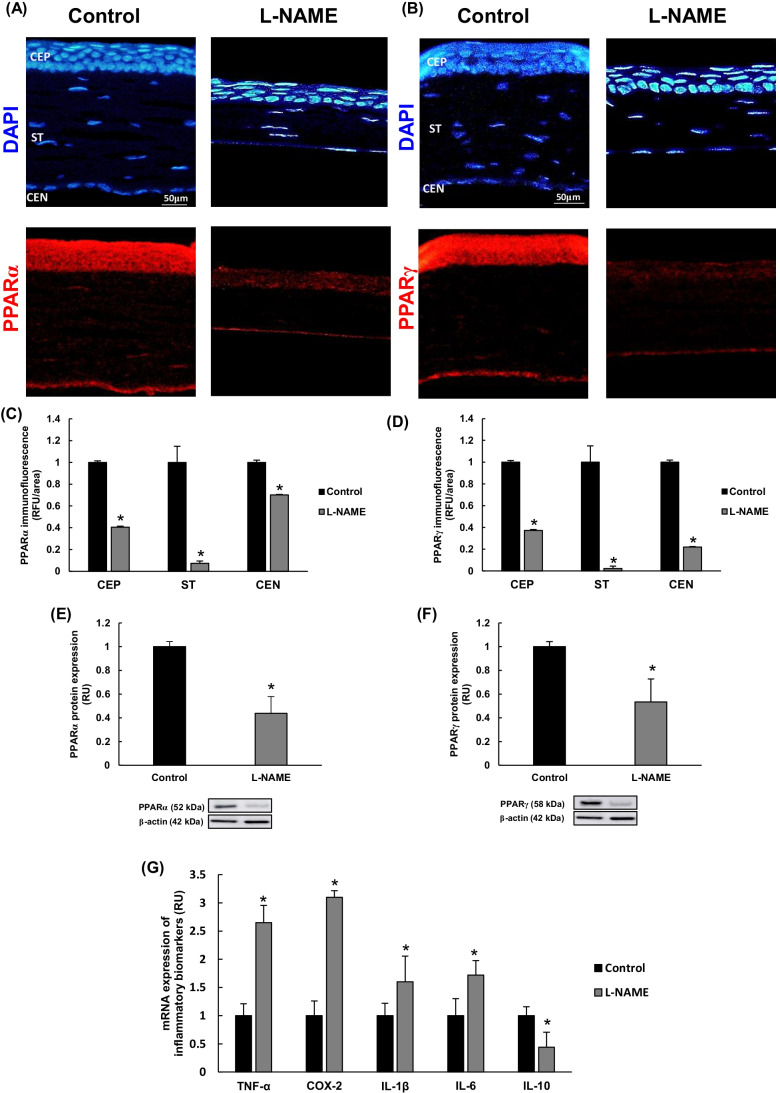
Expression of inflammatory biomarkers in the cornea. **A**–**B** Representative fluorescence signals of (**A**) PPARα and (**B**) PPARγ expression (red color), and nuclei staining with DAPI (blue color) in corneal layers from each experimental group. Magnification: × 10. **C**–**D** Quantitative analyses of fluorescence signals in images represented in **A**–**B**. **E**–**F** PPARα and PPARγ protein expression estimated by Western blotting. **G** mRNA expression of inflammatory biomarkers TNF-α, COX2, IL1-β, IL-6, and IL-10 in cornea homogenates from normotensive (control) and hypertensive (L-NAME) mice. Quantitative fold changes in gene expression were determined relative to the corresponding value for the housekeeping gene GAPDH. Values are expressed as mean ± SEM of seven animals per group. *p < 0.05 vs control. CEN, corneal endothelium; CEP, corneal epithelium; RFU, relative fluorescence units; RU, relative units; ST, stroma

Peroxisome proliferator-activated receptors (PPARs), isoforms PPARα and PPARγ, could be detected by immunofluorescence in CEP, ST, and CEN (Fig. 4A–B). A decrease in PPAR expression was clearly visible in all corneal layers in hypertensive animals. Signal immunofluorescence quantification confirmed downregulation of PPARα (60%, 96%, and 30% reductions compared to the control group in CEP, ST, and CEN, respectively; Fig. 4C), and PPARγ (63%, 98%, and 78% reductions in CEP, ST, and CEN, respectively; Fig. 4D). These results paralleled the relative quantification of PPAR protein expression estimated by Western blotting (Fig. 4E–F).

Concerning cytokines and modulators of inflammation, L-NAME induced the corneal expression of proinflammatory biomarkers TNF-α, COX-2, IL-1β, and IL-6 (~ 2–threefold increase compared to the control group), whereas the anti-inflammatory IL-10 was downregulated (56% reduction) in hypertensive mice (Fig. 4G).

### Modification of corneal collagen deposition in arterial hypertension

**Fig. 5:**
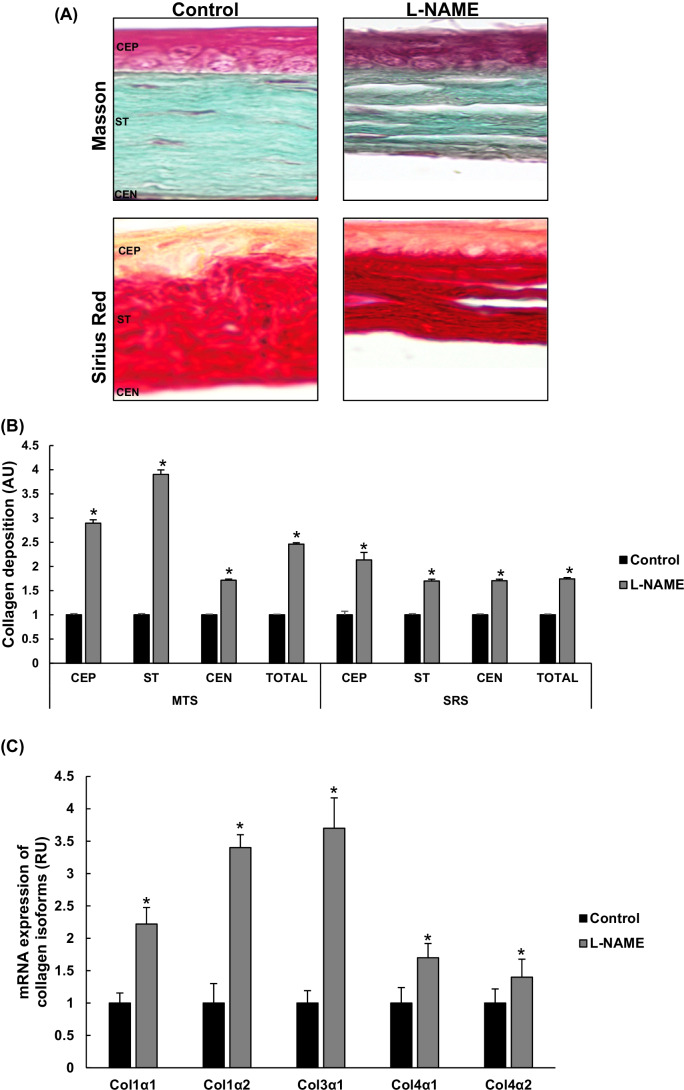
Collagen staining and expression. **A** Representative pictures of corneas stained with Masson’s trichrome (MTS, top panels) and Sirius Red (SRS, bottom panels) in corneal layers from each experimental group. Magnification: × 40. **B** Quantitative analysis of MTS- and SRS-related images in **A**. **C** mRNA expression of collagen isoforms COL1α1, COL1α2, COL3α1, COL4α1, and COL4α2 in cornea homogenates from normotensive (control) and hypertensive (L-NAME) mice. Quantitative fold changes in gene expression were determined relative to the corresponding value for the housekeeping gene GAPDH. Values are expressed as mean ± SEM of seven animals per group. *p < 0.05 vs control. AU, arbitrary units; CEP, corneal epithelium; CEN, corneal endothelium; MTS, Masson’s trichrome staining; RU, relative units; SRS, Sirius Red staining; ST, stroma

Representative pictures of corneal collagen deposition are shown in Fig. 5A. L-NAME-induced hypertension resulted in excess collagen deposition in CEP, ST, and CEN (as observed by darker grayish green and red colors in MTS and SRS images, respectively). Quantification of collagen signals yielded an approximate twofold increase in the corneas of hypertensive mice compared to the control group (Fig. 5B). To confirm whether the visible increase in MTS and SRS staining in the cornea of L-NAME-treated animals was secondary to upregulation of collagen protein, we also quantified the mRNA expression of several collagen isoforms (Fig. 5C). As expected, corneal homogenates from these animals showed overexpression of COL1α1, COL1α2, COL3α1, COL4α1, and COL4α2 ranging ~ 1.5–fourfold increases compared with the respective values found in the normotensive group.

**Fig. 6:**
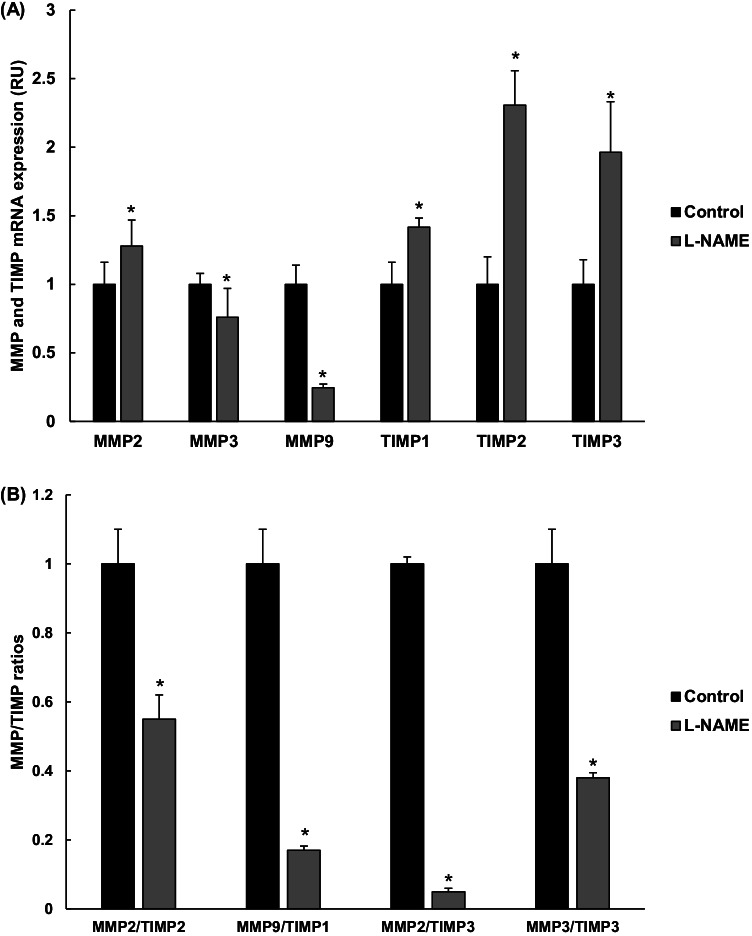
Gene expression of MMPs/TIMPs in the cornea. **A** Gene expression of metalloproteinases (MMP2, MMP3, and MMP9 isoforms) and tissue inhibitors of metalloproteinases (TIMP1, TIMP2, and TIMP3 isoforms) in cornea homogenates from normotensive (control) and hypertensive (L-NAME) mice. **B** The activation status of MMP enzymes in comparison with TIMP expression was estimated from the ratios MMP2/TIMP2, MMP9/TIMP1, MMP2/TIMP3, and MMP3/TIMP3. Quantitative fold changes in gene expression were determined relative to the corresponding value for the housekeeping gene GAPDH. Values are expressed as mean ± SEM of seven animals per group. *p < 0.05 vs control. RU, relative units

Due to the importance of metalloproteinase enzymes (MMP) and their inhibitors (the tissue inhibitors of metalloproteinases, TIMP) in the remodeling of extracellular matrix (ECM), where collagen has an important presence together with other components, the gene expression of different MMP/TIMP isoforms was also determined (Fig. 6A). While an increase of MMP2 (1.3-fold) was found in L-NAME corneas, the expression of MMP3 and MMP9 was reduced by 34% and 76%, respectively, in the same group. On the other hand, these animals presented a remarkable rise of TIMP gene expression (~ 1.5–twofold). The inhibition of MMPs by TIMPs follows a 1:1 stoichiometric ratio [10], where often the preferences are MMP2/TIMP2, MMP9/TIMP1, MMP2/TIMP3, and MMP3/TIMP3. All three ratios were reduced (45%, 83%, 95%, and 62%, respectively) in corneas from L-NAME-treated animals in comparison with normotensive control mice (Fig. 6B), thus suggesting that L-NAME-induced AH increases collagen deposition in the cornea.

## Discussion

Although the negative impact of hypertension on the eye is well documented, specifically in the development of retinopathies [13], the precise mechanisms by which sustained high blood pressure affects the cornea remain unclear. The current data present evidence that, in addition to inducing systemic hypertension, NO depletion in mice produces local oxidative stress in corneal tissue through activation of the NADPH oxidase system, with subsequent downregulation of PPAR expression and release of inflammatory and fibrotic biomarkers. These alterations ultimately affect the morphology of the cornea and lead to increased intraocular pressure, which is described here for the first time in L-NAME-treated rodents and suggests possible mechanisms linking arterial and ocular hypertension.

The experimental animal model of arterial hypertension induced by L-NAME has long provided valuable information on functional, molecular, and pathological mechanisms, as well as possible therapeutic strategies, in the setting of AH [12, 67]. The mechanism of L-NAME action is based on a reduction of nitric oxide (NO) formation due to competitive inhibition of eNOS (the constitutive endothelial isoform of nitric oxide synthase enzyme) and subsequent induction of vasoconstriction, leading to persistent hypertension and end organ damage, including the eye [48–50]. In accordance with previous studies, L-NAME did not affect the animals’ weight gain and dietary intake but produced a significant and sustained increase of SBP from the first week of the treatment [48, 50, 67, 68]. Hence, our experimental approach triggers a situation of severe/malignant hypertension that mimics many of the complications observed in human hypertension, as commonly assumed by specialized institutions and by other authors who use this model to study hypertension-related complications, such as cardiac, renal, or liver damage [1, 26, 29]. Many of the features concerning end-organ damage in rodents treated with L-NAME might be similar to other animal models of hypertension that also present with acutely elevated blood pressure and reduced NO concentration, such as the renovascular or the high salt intake hypertensive models [7, 42]. On the other hand, other models with a different approach to AH pathogenesis, such as the spontaneously hypertensive rat (SHR) [26], might have a different profile in this regard.

The scarce information about corneal damage secondary to AH makes the cornea an attractive ocular component to be studied in this context. Surprisingly, while looking at the eye morphology, we observed that the corneas from hypertensive mice were thinner than those of their normotensive siblings, suggesting an important corneal alteration caused by L-NAME treatment. Corneal thickness has been studied under ocular hypertension (OHT) events, glaucoma, and other corneal diseases [5, 28, 43]. Generally, normal-pressure glaucoma presents with corneal thinning, whereas thicker corneas have been found at initial stages of OHT [53]. Nonetheless, corneal thinning seems to be associated with disease progression and poorer outcomes in both cases, what makes the measurement of corneal thickness an interesting way to detect the severity of the disease [21]. In our experimental conditions, tonometry recordings showed a progressive increase of IOP in L-NAME-treated mice from the middle of the treatment, with remarkable differences between hypertensive and normotensive animals at the end of the experimental period. According to previous studies, an enhanced IOP might be responsible for morphological alterations observed in the cornea of glaucoma hypertensive animals [41], which, in turn, could be associated with aggravated chronic AH. In addition, high IOP values, as typically seen in glaucoma, result in vascular damage with less response to IOP fluctuations; since the cornea, sclera, and lamina cribosa sclerae become thinner under these circumstances [27], one may speculate a similar pattern under AH conditions. Increased IOP values might also be secondary to lower NO bioavailability in corneas from L-NAME-treated animals. The mechanism through which NO can decrease IOP have been previously described; in this regard, NO makes the trabecular meshwork cells and Schlemm’s canal cells more permeable, which increases aqueous humor outflow. However, sustained NO release by the inducible NO synthase (iNOS) is accompanied by an inflammatory state, where the vicious circle of large amounts of NO continuously released in a Ca^2+^-independent way favors the formation of peroxynitrite (ONOO^−^) and exacerbates the inflammatory response [20]. Indeed, iNOS activation and nitrotyrosine formation seems to correlate with visual loss in glaucoma patients [45].

Although some authors claim that systemic blood pressure and IOP run in parallel, other reports show contradictory evidence [54]; in any case, damage to the ocular vasculature is well known in both cases; therefore, vascular alterations related to AH and/or IOP might be responsible for the corneal thinning observed in these animals following mechanisms similar to those attributed to glaucoma [27]. These findings suggest the importance of proper management of AH to avoid corneal diseases associated with a thin cornea, such as corneal ectasias including keratoconus and pellucid marginal degeneration [23, 51]. The current study provides the first evidence of intraocular hypertensive values in the model of AH induced by L-NAME, suggesting possible mechanisms that might link arterial and ocular hypertension.

Oxidative stress is highly related to corneal pathologies, including keratoconus, corneal injuries, and Fuchs endothelial corneal dystrophy [60]; however, the pathways involved in the origin of this oxidative imbalance and subsequent corneal damage are still under research. To the best of our knowledge, here, we present the first evidence of an augmentation of corneal ROS levels in a hypertensive animal model (specifically, L-NAME-treated mice), affecting all corneal layers but especially the CEN. Furthermore, measurements of the activity of NADPH oxidase and NOX isoforms demonstrated a preferential role for NOX4, as inferred from the inhibitory action of GKT136901 (dual NOX1/NOX4 inhibitor) that was much higher in comparison with ML171 (NOX1 inhibitor) and close to that of VAS2870 (pan-inhibitor of NOXes). Additional experiments on the localization and mRNA expression of NOXes confirmed the significant upregulation of NADPH oxidase in corneal layers from L-NAME-treated mice, with a greater effect on NOX4.

The participation of NOX isoforms in pathological corneal events has been studied not only because of its implication on oxidative stress-related damage but also due to its role in inflammation and fibrosis [8, 30]. NOX2 and NOX4 seem to be upregulated after an alkali burn injury of the cornea [18]; therefore, these NOX isoforms might be interesting targets to avoid corneal inflammation and fibrosis, thus limiting the characteristic opacity produced by this injury. NADPH oxidases have also been reported as the major source of O_2_^.−^ in human corneal stromal fibroblasts and epithelial cells [39], and their overexpression in keratoconus is involved in the progression of this corneal disease [2]. All these data suggest that AH might constitute a risk factor for corneal diseases by activating/upregulating NADPH oxidase locally in the corneal layers, which eventually results in an oxidative imbalance, inflammatory, and fibrotic processes in this ocular tissue.

As mentioned above, inflammation and fibrosis play an important role in maintaining the integrity and transparency of the cornea to avoid potentially blinding corneal diseases, including keratoconus, dry eyes, contact lens-related hypoxia, and traumatic and chemical injuries [34]. PPARs play an important role as transcriptional regulators of inflammation, angiogenesis, and fibrosis pathways [4]. The downregulation of these transcription factors contributes to the development and progression of many pathologies with demonstrated role in ocular diseases [69]. In this way, PPAR agonists have been postulated as effective treatments to reduce corneal neovascularization, fibrosis, and inflammation [63]. For instance, in dry eye syndrome, this approach can inhibit inflammatory cytokines such as TNF-α and Il-1β [69]. Moreover, vascular homeostasis is controlled in part by PPARs, which behave as crucial actors in the development of systemic diseases including cardiovascular pathologies, diabetes, cancer, or AH, with specific implications for corneal tissue [24]. In our L-NAME animal model, the significant reduction of PPARα and PPARγ expression found in corneal layers, together with overexpression of well-known proinflammatory biomarkers of corneal diseases (i.e., TNF-α, COX-2, IL-1β, and IL-6 [22]) and downregulation of the anti-inflammatory cytokine IL-10, suggests that, besides the NADPH oxidase family, PPAR-related pathways might be preferential targets in the inflammatory and fibrotic processes that take place in the cornea of hypertensive animals.

The use of MTS and SRS demonstrated excessive collagen deposition in corneal layers of L-NAME-treated mice (mainly in CEP and ST). This could be secondary to a sustained increase in blood pressure levels or a direct effect of altered NO bioavailability in the cornea. In fact, these findings were paralleled by enhanced gene expression of various collagen fibers including type I (COL1α1 and COL1α2), type III (COL3α1), and type IV (COL4α1 and COL4α2) collagen. As mentioned, collagen is the main component of the corneal ECM and a pivotal component to keep the physiological functions of the cornea [44]. Type I collagen is the most abundant collagen in the cornea and its expression can be altered in pathological situations [19]. On the other hand, although the expression of type III collagen in corneal ST is usually weak, an upregulation of this component may be an important marker of stromal matrix remodeling during corneal injury, wound healing, inflammation, and neovascularization [33]. Indeed, the presence of type III collagen in blood vessels makes it a feasible candidate to be affected by AH. In turn, type IV collagen (with predominant expression at the corneal basement and Descemet’s membranes), has also been involved in pathological situations related to corneal opacification [36]. The fact that L-NAME-induced hypertension produced changes in the gene expression of all these collagen fibers suggest that AH-associated inflammation and oxidative imbalance can alter corneal structure with likely consequences for visual function, eventually.

MMPs and TIMPs are widely distributed in many tissues and organs, including the cornea. They are well-known important regulators of fibrotic processes, including in AH [6]. Although it is generally accepted that there is a preferential binding of TIMP2 to MMP2, and TIMP1 to MMP9, other combinations are possible although less likely [9, 71]. For instance, new discoveries are emerging on TIMP3, which is unique among the TIMP family due to its extracellular matrix-binding property and a broad range of inhibitory substrates; TIMP3 can interact with MMP2 and MMP3 [15]. Changes in either MMP or TIMP levels could alter relevant MMP/TIMP ratios and cause a net change in MMP activity [11, 62]. The results of the present study concerning their expression and the ratios MMPs/TIMPs indicate that corneal collagen homeostasis can be modified by AH. Thus, we found an increase in MMP2, TIMP1, TIMP2, and TIMP3 in L-NAME-treated mice, together with lower expression of MMP3 and MMP9. MMP2, MMP3, and MMP9 have been previously reported to be upregulated in inflammatory/fibrotic diseases of the ocular surface, such as corneal ulcer, ocular burn, and corneal neovascularization [25, 47]. However, it is the expression of metalloproteinase inhibitors that seems to be crucial for the development of pathological corneal events such as keratitis or keratoconus [37, 55]. The participation of TIMPS in corneal diseases seems so important that peptidomimetics have been evaluated as a potential therapeutical strategy to decrease corneal tissue damage and improve clinical outcomes of infectious keratitis [37]. When MMP/TIMP ratios were analyzed in our experimental conditions, MMP2/TIMP2, MMP9/TIMP1, MMP2/TIMP3, and MMP3/TIMP3 ratios were reduced, indicating an overall inhibition of MMPs in the cornea. Downregulation of some MMPs accompanied by increased TIMP expression has been reported by other authors in hypertensive animal models [65]. These findings have been previously explained as an inability of the body to degrade the excessive collagen deposition observed in severe hypertension [3], contributing to fibrogenesis of the hypertensive target organ. Recent experiments carried out in our laboratory showed a similar MMP/TIMP pattern in the retina of L-NAME hypertensive mice (unpublished results), which supports the notion that hypertension-dependent fibrotic processes occur at different ocular layers. Either way, all these findings sustain the need to monitor ocular function and check for potential fibrotic processes in the context of hypertensive eye diseases.

## Conclusion

The current study provides the first evidence that L-NAME-induced arterial hypertension presents with corneal morphological changes, which might be secondary to sustained elevation of IOP and systemic blood pressure values, or a direct effect of altered NO bioavailability in the cornea. In this rodent model of hypertension secondary to NO depletion, the animals showed clear signs of oxidative stress, inflammation, and fibrotic events by stimulation of the NADPH oxidase system, with altered PPAR function, overexpression of major proinflammatory biomarkers, and, eventually, excessive collagen deposition on the cornea. Our observations reinforce the importance of monitoring ocular tissues, specifically the cornea, in the setting of hypertension-related eye diseases.
